# Characterizing fatty acid oxidation genes in *Drosophila*

**DOI:** 10.1093/g3journal/jkaf139

**Published:** 2025-06-16

**Authors:** Juliana Geronazzo, Abigail Heimerl, Linnea Lindell, Skye McCrimmon, Clara Stormer, Brooke Horvai, Ian P Johnson, Tia M Peterson, Jocelyn Zuckerman, Anna I Scott, Meredith M Course

**Affiliations:** Department of Molecular Biology, Colorado College, 14 E Cache La Poudre St, Colorado Springs, CO 80903, USA; Department of Molecular Biology, Colorado College, 14 E Cache La Poudre St, Colorado Springs, CO 80903, USA; Department of Molecular Biology, Colorado College, 14 E Cache La Poudre St, Colorado Springs, CO 80903, USA; Department of Molecular Biology, Colorado College, 14 E Cache La Poudre St, Colorado Springs, CO 80903, USA; Department of Molecular Biology, Colorado College, 14 E Cache La Poudre St, Colorado Springs, CO 80903, USA; Department of Laboratory Medicine and Pathology, University of Washington, 1959 NE Pacific St, Box 357470, Seattle, WA 98195, USA; Department of Molecular Biology, Colorado College, 14 E Cache La Poudre St, Colorado Springs, CO 80903, USA; Department of Molecular Biology, Colorado College, 14 E Cache La Poudre St, Colorado Springs, CO 80903, USA; Department of Molecular Biology, Colorado College, 14 E Cache La Poudre St, Colorado Springs, CO 80903, USA; Department of Laboratory Medicine and Pathology, University of Washington, 1959 NE Pacific St, Box 357470, Seattle, WA 98195, USA; Department of Laboratories, Seattle Children’s Hospital, FB.4.510, 4800 Sand Point Way NE, Seattle, WA 98105, USA; Department of Molecular Biology, Colorado College, 14 E Cache La Poudre St, Colorado Springs, CO 80903, USA

**Keywords:** *Drosophila*, fatty acid oxidation, inborn errors of metabolism, β-oxidation, electron transfer, CRISPR-Cas9, *Arc42*, *CG4860*, *Mcad*, *Mtpα*, *Etf-QO*, *CG7834/Etfb*

## Abstract

In this study, we leverage the power and tractability of *Drosophila* genetics to better understand the molecular mechanisms underlying a group of rare genetic diseases known as fatty acid oxidation disorders. We use CRISPR-Cas9 to generate mutations in 6 putative fatty acid oxidation genes in *Drosophila*, then analyze the phenotypes and acylcarnitine profiles of these flies. We find that while *Arc42* and *CG4860* are both predicted orthologs of human *ACADS*, only *Arc42* loss of function mirrors the acylcarnitine profile of *ACADS* loss of function. Acylcarnitine profiles also support our previous identification of *Mcad* as the likely *ACADM* ortholog, and reveal the deleterious effects of a single codon deletion in *Mtpα* (the predicted human *HADHA* ortholog). Finally, we observe that loss of function in *Etf-QO* and in *CG7834*—predicted orthologs of human *ETFDH* and *ETFB*, respectively—is homozygous lethal in flies. Producing animal models like these will enable new approaches to studying fatty acid oxidation disease progression, symptomatic variability, and therapeutic intervention.

## Introduction

Inborn errors of metabolism (IEMs) are devastating genetic diseases that can manifest at any age, typically in response to physiological stress. Because IEMs account for a notable percentage of sudden infant death syndrome cases (van Rijt *et al*. 2016), many are included in newborn screening. Fatty acid oxidation disorders (FAODs) are a subtype of IEM caused by the inability to break down fatty acids, all of which are inherited as autosomal recessive conditions. Despite known biochemical mechanisms, there is significant variability in disease severity, and the pathophysiology is still not fully understood (Houten and Wanders 2010). Therapy is primarily dietary management and treatment of symptoms, the effectiveness of which varies by disease. Currently, FAODs are identified by characteristic acylcarnitine profiles, measured in blood by tandem mass spectrometry (Miller *et al*. 2021).

**Fig. 1.**
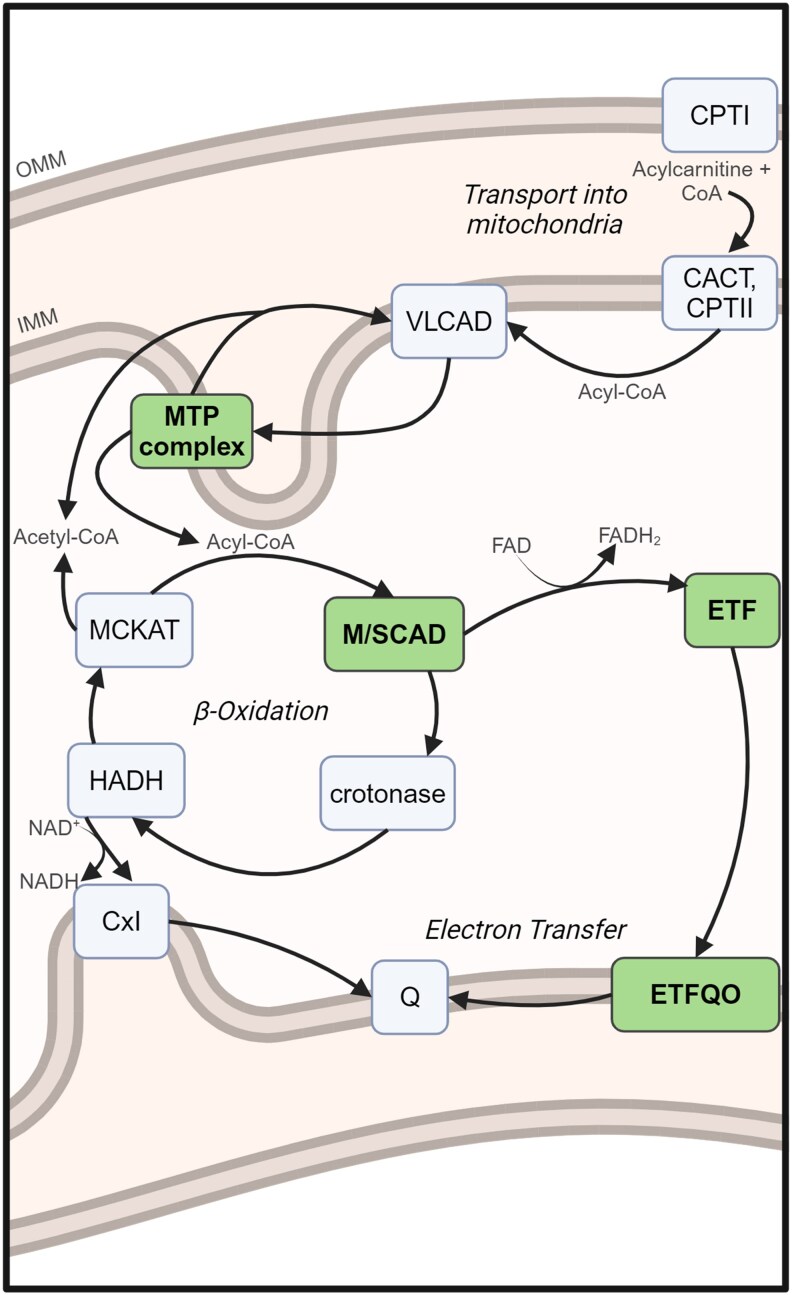
Schematic of the mitochondrial fatty acid oxidation pathway in humans. Enzymes in this study are in bold. Some substrates and products have been omitted for simplicity. CACT, carnitine acylcarnitine translocase; CoA, coenzyme A; CPT, carnitine palmitoyltransferase; Cx I, complex I of respiratory chain; ETF, electron transfer flavoprotein; ETFQO, ETF ubiquinone oxidoreductase; FAD, flavin adenine dinucleotide; FADH_2_, reduced FAD; HADH, 3-hydroxyacyl-CoA dehydrogenase; IMM, inner mitochondrial membrane; MCKAT, medium-chain ketoacyl-CoA thiolase; MTP, mitochondrial trifunctional protein; M/SCAD, medium-chain/short-chain acyl-CoA dehydrogenase; NAD^+^, nicotinamide adenine dinucleotide; NADH, reduced NAD; OMM, outer mitochondrial membrane; Q, ubiquinone; VLCAD, very-long-chain acyl-CoA dehydrogenase. Adapted from Saudubray *et al*. (2016). Created in BioRender. Heimerl (2025). https://BioRender.com/o91q148.

Fatty acids, glucose, and amino acids are the building blocks of macromolecules and major sources of energy in the body. Glucose acts as the primary source of energy through cellular respiration and glycolysis; however, when cells are depleted of glucose (e.g. stressful conditions), they rely on fatty acid metabolism. Mitochondrial fatty acid oxidation (FAO) occurs in 3 steps: (1) fatty acid transport into the mitochondria via the exchange of coenzyme A (CoA) with carnitine, (2) β-oxidation, in which acyl-CoAs are shortened by 2 carbons in a spiral pathway, and (3) electron transfer, in which the reduced electron carriers ferry their electrons to the electron transport chain (Fig. 1) (Saudubray *et al.* 2016).

As part of mitochondrial metabolism, FAO is relatively conserved across species, opening the possibility of studying FAODs in model organisms. Several mouse models of FAODs exist, though the mouse phenotype does not always match the human symptomology (Houten and Wanders 2010; Gaston *et al*. 2023). Our previous work showed that a *Drosophila* model of one of these deficiencies [medium-chain acyl-CoA dehydrogenase (MCAD) deficiency] recapitulated the metabolic patterns observed in human patients (Course *et al*. 2018). Buoyed by the similarities between human and *Drosophila* acylcarnitine profiles, we were interested in continuing to test putative *Drosophila* orthologs of human FAO genes for functional conservation.

*Drosophila* is one of the most genetically tractable model organisms, with high fecundity, rapid reproduction, low costs, and easily controlled environmental conditions, making them an ideal model for studying FAOD variability (Hales *et al*. 2015). In this study, we explore putative orthologs of human FAO genes in *Drosophila* to establish them as robust models of FAODs. To this end, we used CRISPR-Cas9 to mutate 6 genes in *Drosophila*, summarized in Table 1 and Fig. 1, then observed their resultant phenotypes. We find that while *Arc42* and *CG4860* are both predicted orthologs of human *ACADS*, the acylcarnitine profile for *Arc42* loss of function mimics that of *ACADS* loss of function, while that of *CG4860* does not. Acylcarnitine profiles also recapitulate our previous finding that *Mcad* is the likely ortholog of human *ACADM*, and show the deleterious effects of a single codon deletion in *Mtpα* (the predicted ortholog of human *HADHA*). Finally, we observe that loss of function for *Etf-QO* as well as for *CG7834—*predicted orthologs of human *ETFDH* and *ETFB*, respectively—is homozygous lethal in flies.

**Table 1.** Genes observed in this study.

Human symbol	Human name	Fly symbol	Fly name
β-Oxidation			
* ACADS*	acyl-CoA dehydrogenase short chain	*Arc42*	Activator-recruited cofactor subunit 42
* ACADS*	acyl-CoA dehydrogenase short chain	*CG4860*	(*none*)
* ACADM*	acyl-CoA dehydrogenase medium-chain	*Mcad*	Medium-chain acyl-CoA dehydrogenase
* HADHA*	hydroxyacyl-CoA dehydrogenase trifunctional multienzyme complex subunit alpha	*Mtpα*	Mitochondrial trifunctional protein α subunit
Electron transfer			
* ETFDH*	electron transfer flavoprotein dehydrogenase	*Etf-QO*	Electron transfer flavoprotein-ubiquinone oxidoreductase
* ETFB*	electron transfer flavoprotein subunit beta	*CG7834/Etfb*	Electron transfer flavoprotein beta subunit

## Methods and materials

### Generation of *Drosophila* mutations

All *Drosophila* mutations were generated using reagents and protocols from the DRSC/Transgenic RNAi Project (TRiP) Functional Genomics Resources at Harvard Medical School (Zirin *et al*. 2020). Briefly, a nanos-Cas9 stock was crossed to a TRiP-KO stock containing the target sgRNA (Table 2) (Ren *et al*. 2013). Male F1 progeny containing both transgenes were collected and crossed to the appropriate balancer strain *en masse* (Table 2). The F2 progeny were collected and individually crossed to the appropriate balancer strain. Finally, the balanced F3 progeny were collected while transgenes were eliminated using a visible marker. Control lines were made in the same manner, including balancing for each target chromosome, using the fly that the sgRNAs were injected into (Table 2). All flies were obtained from the Bloomington *Drosophila* Stock Center (BDSC) and maintained at 25°C on Nutri-Fly Bloomington Formulation food (Flystuff) with propionic acid. FlyBase was used throughout this study (Öztürk-Çolak *et al*. 2024).

**Table 2.** *Drosophila* stocks used in study.

Relevant fly target	Genotype	BDSC RRID^a^
*Arc42*	y[1] sc[*] v[1] sev[21]; P{y[ + t7.7] v[ + t1.8] = TKO.GS00546}attP40	BDSC_76377
*CG4860*	y[1] sc[*] v[1] sev[21]; P{y[ + t7.7] v[ + t1.8] = TKO.GS03434}attP40	BDSC_83752
*CG7834/Etfb*	y[1] sc[*] v[1] sev[21]; P{y[ + t7.7] v[ + t1.8] = TKO.GS01855}attP40	BDSC_79795
*Etf-QO*	y[1] sc[*] v[1] sev[21]; P{y[ + t7.7] v[ + t1.8] = TKO.GS01805}attP40	BDSC_79768
*Mcad*	y[1] sc[*] v[1] sev[21]; P{y[ + t7.7] v[ + t1.8] = TKO.GS00853}attP40	BDSC_77065
*Mtpα*	y[1] sc[*] v[1] sev[21]; P{y[ + t7.7] v[ + t1.8] = TKO.GS00727}attP40	BDSC_77201
*nanos-Cas9*	y[1] sc[*] v[1] sev[21]; P{y[ + t7.7] v[ + t1.8] = nanos-Cas9.R}attP2	BDSC_78782
*Balancer used for chr. II*	y[1] sc[*] v[1] sev[21]; In(2LR)Gla, wg[Gla-1] PPO1[Bc]/CyO	BDSC_35781
*Balancer used for chr. III*	y[1] sc[*] v[1] sev[21]; Dr[1] e[1]/TM3, Sb[1]	BDSC_32261
*Injection line used to generate controls*	y[1] v[1]; P{y[ + t7.7] = CaryP}Msp300[attP40]	BDSC_36304

^*a*^BDSC Research Resource Identification.

### PCR, gel electrophoresis, and purification

To confirm mutations, PCR was performed on DNA extracted from single flies using OneTaq Quick-Load 2× Master Mix with Standard Buffer (New England Biolabs) following the manufacturer's protocol and using primers described in Table 3. To confirm successful PCR amplification, samples were run on a 1% w/v agarose gel made with 1× TAE and SYBR Safe DNA Gel Stain (Invitrogen), alongside a 1 kb Plus DNA Ladder for Safe Stains (New England Biolabs). To prepare for sequencing, PCR products were purified using a Monarch PCR and DNA Cleanup Kit (New England Biolabs), following the manufacturer's protocol.

**Table 3.** Primers used to study each gene.

Fly gene targeted	Primers (5′→3′)	
	Forward	Reverse
*Arc42*	CTCTGGTCACACTGTCCATTT	CATCTGGCGGATCTGTTTCT
*CG4860*	AGGATAATCGCTGGTGGAAAC	GCACCCAGGTACAGGTTATTC
*CG7834*	TGGAGTTAAGCGTGTGATCG	TCCAGGCCACCATCAATTT
*Etf-QO*	CGGGTCTAAAGTCCAACTTGT	CCTCAACACAACGCTCCATA
*Mcad*	CCACATTCAAATGGCGTTCC	GAGATCCAGGCCACCAATATC
*Mtpα*	GATCAAGATCGACTCACCCAA	AGTTTGGTCTTGCTGTCCTT

### Sanger sequencing and analysis

Purified samples were sent to Genewiz (Azenta Life Sciences) for Sanger sequencing. Sequences were assessed for quality and aligned to the wild-type *Drosophila* genome using Benchling (https://benchling.com). Alignment revealed deletions present in the mutant flies, all located within the sgRNA target regions. Sequencing of each sample was repeated 3 times to ensure that the same mutation was present each time. Chromatogram images used in this paper were made using SnapGene (Dotmatics).

### Western blotting

To confirm protein knockout in the *Mcad* mutant flies, one 1–4-day old male and one 1–4-day old female fly per sample were homogenized in RIPA buffer (Thermo Scientific) at a ratio of 10 µL per fly. Samples were homogenized with pellet pestles, then centrifuged at 13,000 rpm at 4°C for 30 min to remove debris. Supernatant was run on an Any kD Mini-Protean TGX Precast Protein Gel (Bio-Rad) along with HeLa whole cell lysate (Abcam ab29545). The proteins were immunoblotted with anti-MCAD antibody at 1:500 (Abcam ab110296), followed by HRP-conjugated goat anti-mouse IgG (Jackson ImmunoResearch 115035166) at 1:5000. Total protein labeling was accomplished using the No-Stain Protein Labeling Reagent (Invitrogen), following the manufacturer's protocol. Chemiluminescent visualization was performed using SuperSignal West Pico Plus Chemiluminescent Substrate (Thermo Scientific) and imaged on an iBright CL1500 Imaging System (Invitrogen).

### Acylcarnitine profiles

All flies were flipped into fresh food every 3 days and assayed at 6 days old. For the starvation cohort, flies were transferred to a vial containing only water for the final 24 h before extraction. Six flies per sample (3 female and 3 male) were homogenized in 3:1 methanol:acetonitrile (10 µL per fly). Samples were then centrifuged at 10,000 rpm for 2 min to remove debris. 45 µL of supernatant was removed and placed into a clean centrifuge tube, which was then left open in a 37°C incubator for 1 h, until all the liquid had evaporated for stability during shipping.

Sample analysis was performed similarly to previous work (Course *et al*. 2018). Controls (high, low, and negative) were included with each batch of acylcarnitines using 25 μL of sample. Isotope-labeled internal standards (Cambridge Isotope) in acetonitrile and 0.4% formic acid (Sigma) were added directly to each sample to a total volume of 225 μL. Samples were vortexed and centrifuged for 5 min at 13,000 rpm. Supernatant was decanted into glass reaction vials and dried for at least 45 min under nitrogen. Acylcarnitines were resuspended and derivatized in 100 μL of 3N HCl in butanol (Regis Technologies Inc.) and incubated at 50°C for 15 min. Samples were dried again under nitrogen. 200 μL of hexane was added to each vial, vortexed, then inverted to pour out hexane and remove phospholipids. Samples were dried to completion under nitrogen to ensure hexane removal. Finally, samples were resuspended in 100 μL of 80% acetonitrile and transferred to glass injection vials for analysis on a tandem mass spectrometer (Waters Xevo TQ). Injection volume was set at 10 μL. The acylcarnitine profile was collected in positive mode using precursor scanning of *m/z* 85. Data were acquired and quantified using MassLynx version 4.1 and NeoLynx Browser version 4.1.

### Protein alignment

Protein sequences were obtained from UniProt (The UniProt Consortium 2025). Alignments were performed using Clustal Omega (Madeira *et al*. 2024). SUPERFAMILY protein domains were identified using InterPro (Blum *et al*. 2025).

### Statistical analysis

Acylcarnitine profiles were statistically analyzed using One-Way ANOVA followed by Tukey's multiple comparisons. Analyses were performed using Prism version 10.4.0 for Windows (GraphPad Software, Boston, Massachusetts USA, www.graphpad.com).

## Results

### Identifying target genes

To determine which *Drosophila* genes were the predicted orthologs of 16 human FAOD genes (Saudubray *et al.* 2016), we used the *Drosophila* RNAi Screening Center (DRSC) Integrative Ortholog Prediction Tool (DIOPT; Supplementary Table 1) (Hu *et al*. 2011). All genes had at least 1 predicted ortholog in *Drosophila*, with the exception that *ACADVL* and *ACAD9* were both predicted to be orthologs of fly *CG7461*; this is unsurprising, given that in humans these are considered homologous (Saudubray *et al.* 2016). We then cross-referenced the moderate-to-high scoring genes from this list to FlyBase (Öztürk-Çolak *et al*. 2024) to confirm predicted function. Finally, we cross-referenced this list to the flies carrying sgRNAs targeting these genes available through the DRSC/TRiP Functional Genomics Resources in vivo CRISPR fly stocks (Zirin *et al*. 2020), of which there were 13 This list included for the best predicted DIOPT score for each gene, with the exception of predicted orthologs of *SLC25A20*, *CPTII*, and *NADK2*; again, the predicted ortholog for *ACAD9* and *ACADVL* was the same. We obtained 2 different lines carrying sgRNAs targeting predicted orthologs of *ACADS*: for *CG4860* and *Arc42*. Ultimately, 6 lines plus 2 control lines were successfully crossed and screened for mutations. The other 7 remain untested for technical reasons. We elected to generate knockouts instead of knockdowns because many of these disorders' symptomologies are masked by residual activity of the enzyme.

### Deletion mutations generated by CRISPR-Cas9

**Fig. 2.**
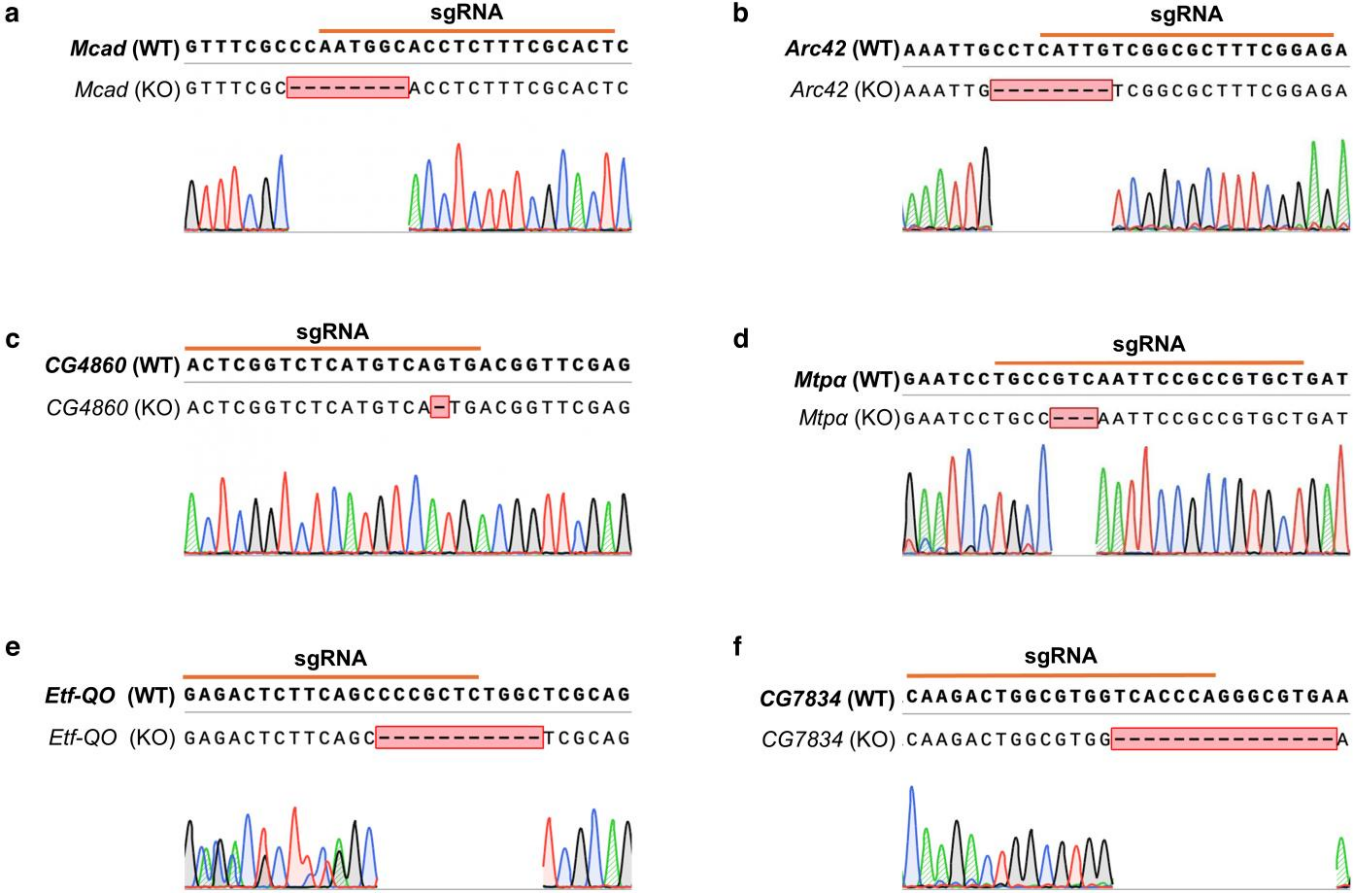
Validation of deletions in mutated flies. Sanger sequencing chromatograms show deletion region in a) *Mcad*, b) *Arc42*, c) *CG4860*, d) *Mtpɑ*, e) *Etf-QO*, and f) *CG7834.* Barred in orange is the sgRNA target region. The first row is the wild-type sequence of the gene of interest; the second row is the aligned mutated sequence. The deletions present in the mutated flies are boxed in their respective KO sequences.

**Fig. 3.**
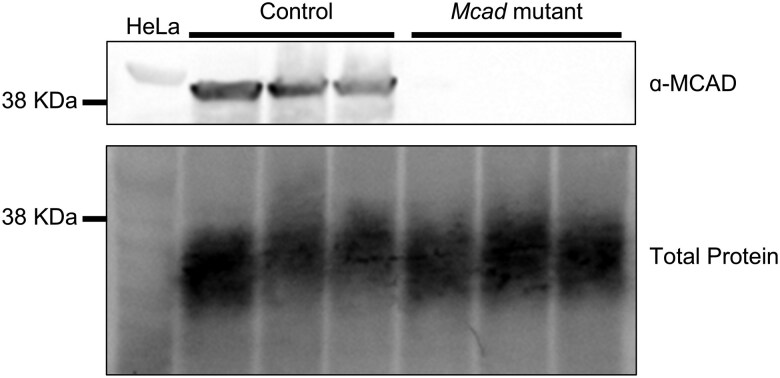
MCAD protein is absent in *Mcad* mutant flies. Western blotting of HeLa lysate, control fly lysate, and *Mcad* mutant fly lysate, probed with ɑ-MCAD antibody (top). Loading for the same blot was confirmed using a total protein label (bottom). KDa is kilodaltons. Raw images are provided in Supplementary Fig. 1.

The available flies carrying sgRNAs targeting our genes of interest were crossed to a nanos-Cas9 stock, which expresses Cas9 in the germline (Ren *et al*. 2013). After selection and balancing, we generated 5 fly lines with early frameshift mutations, in: *Mcad*, *Arc42*, *CG4860*, *Etf-QO*, and *CG7834* (Fig. 1; Table 1). Mutations were confirmed via PCR and Sanger sequencing, and all consisted of deletions ranging from 1 to 15 base pairs, all in the sgRNA target region (Fig. 2). Because the sgRNAs all targeted sequences in exon 2 and the recovered mutations were predicted to cause frameshifts, we predicted loss of function for each gene's encoded protein. Another gene, *Mtpα*, was also successfully mutated using this process, but the resulting 3 base pair deletion led to the removal of a single valine (Fig. 2d). Successful knockout of MCAD protein was confirmed via western blot: the MCAD protein was present in all control flies, and not in the mutant flies (Fig. 3; Supplementary Fig. 1). We performed western blots for the other mutant flies; however, commercially available antibodies were only verified to react with vertebrate proteins and did not detect the proteins in flies, thus precluding our ability to verify protein knockout.

After successfully mutating these genes in flies, we tested whether they shared functional conservation with their predicted human ortholog by acylcarnitine analysis. Acylcarnitine profiles are the primary method used to identify human FAODs. Briefly, tandem mass spectrometry quantifies a variety of acylcarnitines in a sample, and these are compared with established normal ranges for each metabolite. Each FAOD has a stereotypical acylcarnitine profile in humans (Miller *et al*. 2021), and we compared the *Drosophila* acylcarnitine profiles to those of humans with the predicted ortholog affected.

### *Mcad* loss of function mimics that of *ACADM*

**Fig. 4.**
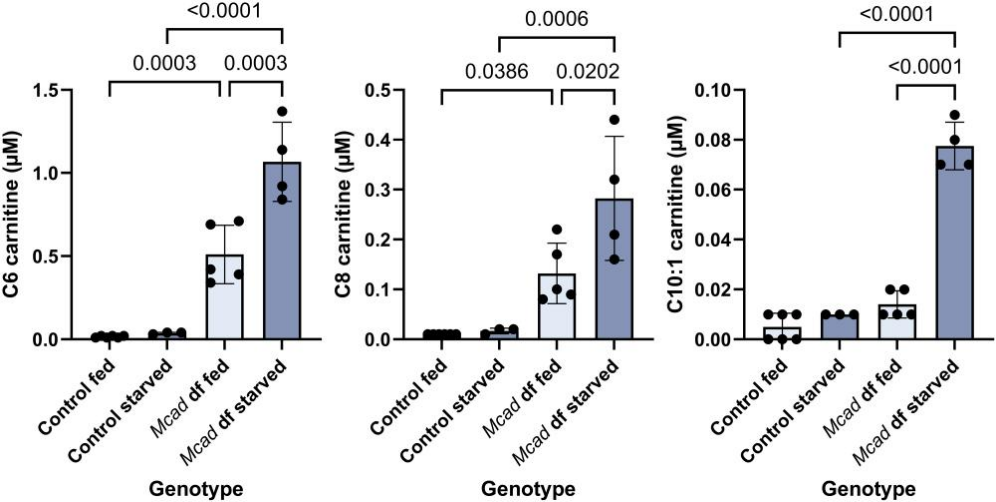
Medium-chain acylcarnitines are elevated in *Mcad* mutant flies. Levels of C6, C8, and C10:1 acylcarnitines in fed and starved *Mcad* mutant flies and controls. “Df” means “deficient.” Error bars are standard deviation. Relevant significant differences with *P*-values are given according to one-way ANOVA, followed by Tukey's multiple comparisons.

*Mcad*, previously known as *CG12262*, was identified in our previous work as being the likely ortholog of human *ACADM* (Course *et al*. 2018), and was re-named *Mcad* because of it. It was therefore expected that our new *Mcad* deficient flies would recapitulate the acylcarnitine profile found in human individuals with MCAD deficiency, which includes significant elevations of acylcarnitines C6, C8, and C10:1 compared with controls. This pattern was observed in our homozygous *Mcad* deficient flies, and, as expected, is exacerbated in starved animals vs fed (starved means water only was provided for the final 24 h before acylcarnitine extraction) (Fig. 4). The C10:1 elevation only achieved significance under the starved condition, which is consistent with the fact that it is the least abnormal metabolite in MCAD deficiency in humans. While not a novel finding, this new fly knockout data validates the previous study, and serves as a positive control for the rest of this work.

It is worth noting that in these and all other samples, we observed a significant elevation of C3 (propionylcarnitine) in all fed flies as compared to starved flies. This elevation is due to the propionic acid that is commonly used in fly food to prevent fungal growth (Ashburner and Roote 2007). This finding was consistent across all samples, regardless of genotype, and was not expected to affect other acylcarnitine levels (Supplementary Table 2).

### *Arc42* loss of function mimics that of *ACADS*

**Fig. 5.**
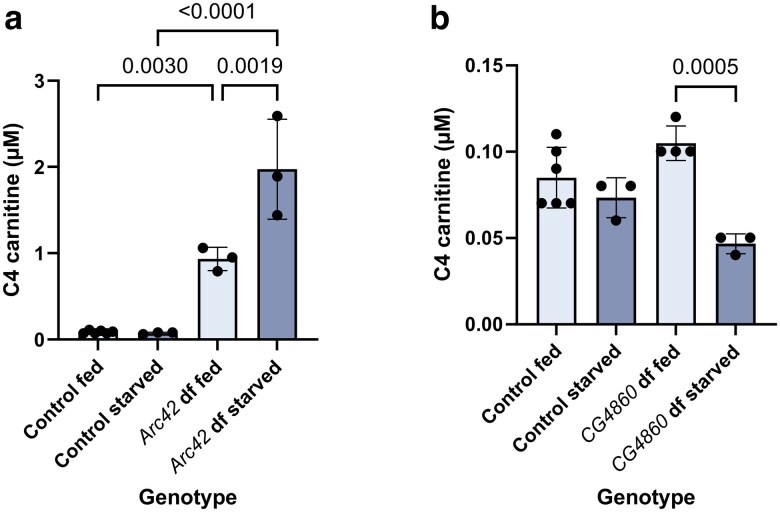
Short chain acylcarnitines are elevated in *Arc42* but not *CG4860* mutant flies. Levels of C4 acylcarnitines in fed and starved a) *Arc42* and b) *CG4860* mutant flies and controls. “Df” means “deficient.” Error bars are standard deviation. Relevant significant differences with *P*-values are given according to one-way ANOVA, followed by Tukey's multiple comparisons.

**Fig. 6.**
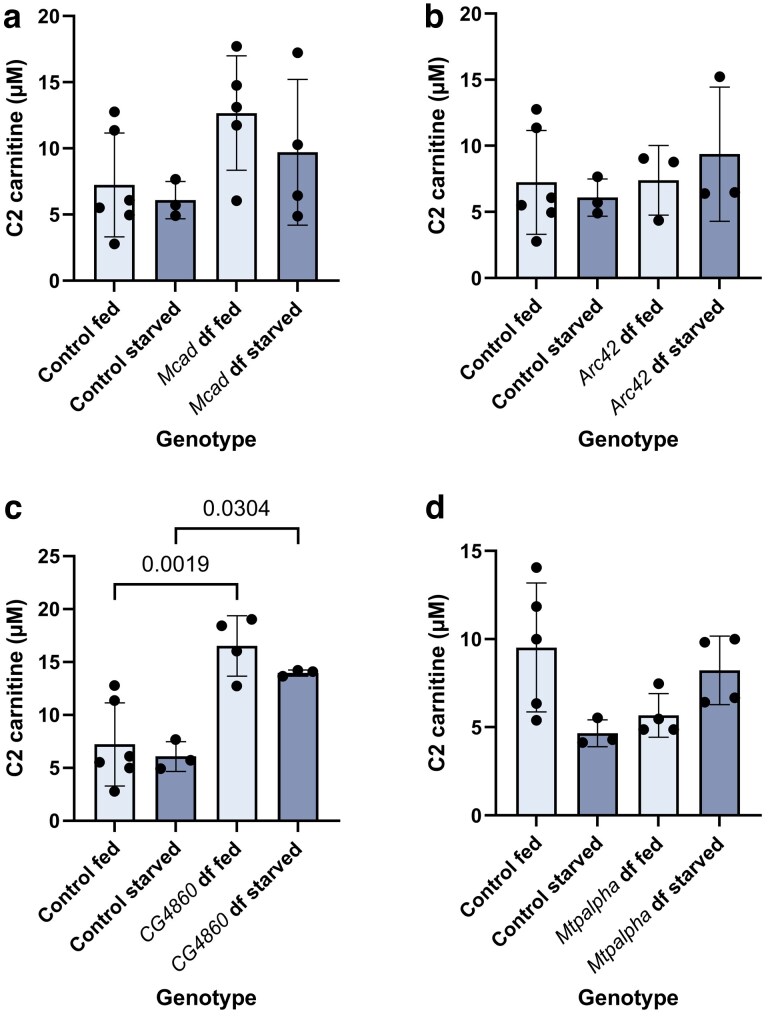
Acylcarnitine C2 levels across homozygous mutant flies. Shown for a) *Mcad*, b) *Arc42*, c) *CG4860*, and d) *Mtpɑ.* “Df” means “deficient.” Error bars are standard deviation. Relevant significant differences with *P*-values are given according to one-way ANOVA, followed by Tukey's multiple comparisons.

Two different fly genes were predicted by DIOPT to be orthologs of human *ACADS*: *Arc42* and *CG4860* (Supplementary Table 1). While *CG4860* received a lower prediction score from the algorithm, they are both annotated on FlyBase as predicted orthologs of *ACADS*. In human individuals with SCAD (short-chain acyl-CoA dehydrogenase) deficiency, the stereotypical acylcarnitine profile is dominated by elevated C4 (butyrylcarnitine) (Miller *et al*. 2021). C4 was elevated in the homozygous *Arc42* mutant flies, and this elevation was exacerbated in the starved condition, as expected (Fig. 5a). In contrast, we did not observe an elevation of acylcarnitine C4 in the homozygous *CG4860* mutant flies as compared to controls in either condition (Fig. 5b). Instead, we observed a significantly *lower* concentration of C4, and a significant elevation of C2 (acteylcarnitine). This is the only genotype for which we observed an elevation in C2 (Fig. 6).

### A single codon deletion disrupts *Mtpα* function

The fly gene *Mtpα* was predicted by DIOPT to be orthologous to human *HADHA*. Pathogenic variants in *HADHA*, as well as in *HADHB*, cause long-chain hydroxyacyl-CoA dehydrogenase (LCHAD) deficiency and trifunctional protein (TFP) deficiency. In these enzyme deficiencies, acylcarnitines C16, C18, C18:2, C18:1, C14–OH, C16–OH, and C18–OH are elevated.

**Fig. 7.**
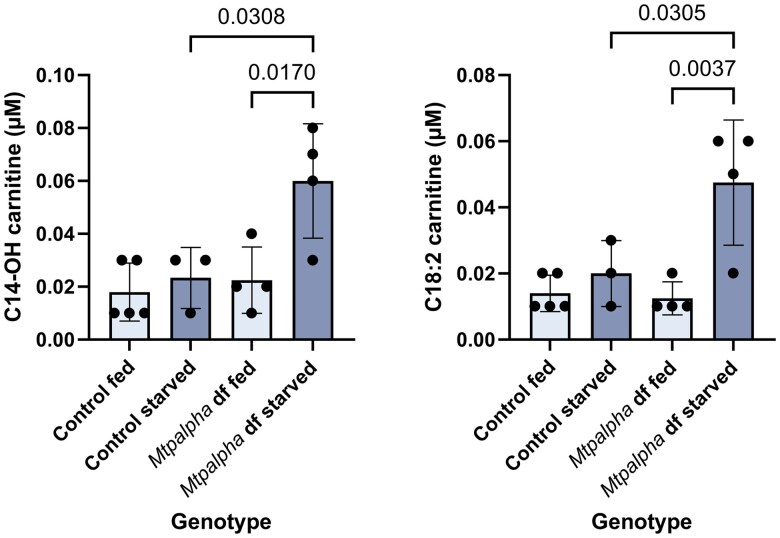
Some long chain acylcarnitines are elevated in starved *Mtpɑ* mutant flies. Levels of C14-OH and C18:2 acylcarnitines in fed and starved *Mtpɑ* mutant flies and controls. “Df” means “deficient.” Error bars are standard deviation. Relevant significant differences with *P*-values are given according to one-way ANOVA, followed by Tukey's multiple comparisons.

The deletion induced by CRISPR-Cas9 in our flies removed exactly 1 codon encoding a valine within exon 2 (Fig. 2d). Despite not being a frameshift mutation, we were still interested to see whether this change would affect protein function, especially as the amino acid at this location in humans is an isoleucine (Supplementary Figs. 2 and 3). While not identical, valine and isoleucine are both branched chain amino acids, and are structurally similar. Acylcarnitine profiles of these flies and their controls showed that C14–OH and C18:2 are significantly elevated under starvation conditions (Fig. 7).

### *Etf-QO* and *CG7834* disruptions are both homozygous lethal in *Drosophila*

In addition to studying 4 genes implicated in β-oxidation, we studied 2 genes involved in electron transfer: *Etf-QO* and *CG7834*. Knocking out each gene turned out to be homozygous lethal in *Drosophila*. We did run acylcarnitine profiles on the heterozygous *Etf-QO* flies, but as expected, did not observe the numerous acylcarnitine elevations that are present in humans with deficiencies in the orthologous gene (Supplementary Table 2). In humans with electron transfer deficiency, called multiple acyl-coA dehydrogenase (MAD) deficiency, we expect to see wide-ranging and severe alteration of acylcarnitines. Because our flies still have 1 functional copy of *Etf-QO* in this example, it is not surprising that it does not recapitulate the acylcarnitine profile of a human with an autosomal recessive and therefore homozygous disorder.

## Discussion

In this study, we used CRISPR-Cas9 to generate mutations in *Drosophila* in 6 putative FAO genes: *Mcad*, *Arc42*, *CG4860*, *MTPα*, *Etf-QO*, and *CG7834* (Fig. 1; Table 1). We first showed that CRISPR-Cas9 successfully removed 1–15 base pairs in each fly, leading to an early frameshift mutation and therefore a predicted knockout in 5, and the removal of 1 amino acid in 1 (Fig. 2; Table 4). In the *Mcad* mutant flies, we were additionally able to confirm protein knockout via western blot (Fig. 3; Supplementary Fig. 1).

**Table 4.** Summary of study results.

Fly symbol	Closest human symbol	Mutation generated	Observed phenotype
*Arc42*	*ACADS*	8 bp deletion, predicted frameshift	Elevated C4 acylcarnitine (Fig. 5a)
*CG4860*	*ACADS*	1 bp deletion, predicted frameshift	Lower C4 acylcarnitine, elevated C2 acylcarnitine
			(Figs. 5b and 6)
*Mcad*	*ACADM*	8 bp deletion, predicted frameshift, confirmed protein knockout	Elevated C6, C8, and C10:1 acylcarnitine (Fig. 4)
*Mtpα*	*HADHA*	3 bp deletion, predicted removal of one codon	Elevated C14-OH and C18:2 acylcarnitine (Fig. 7)
*Etf-QO*	*ETFDH*	11 bp deletion, predicted frameshift	Homozygous lethal
*G7834/Etfb*	*ETFB*	15 bp deletion, predicted frameshift	Homozygous lethal

We then used acylcarnitine analysis to measure the acylcarnitine levels in each of the fly mutants, as compared to controls. For the *Mcad* knockout flies, we observed the C6, C8, and C10:1 elevations characteristic of MCAD deficiency in humans (Fig. 4) (Course *et al*. 2018). For the *Arc42* mutants, we observed the C4 elevation characteristic of SCAD deficiency in humans (Fig. 5a), but did not observe this pattern for *CG4860* (Fig. 5b). For the *MTPα* mutants, we observed that just 1 amino acid deletion led to elevations in some of the acylcarnitines that are elevated in LCHAD and TFP deficiencies (Fig. 7). Finally, for the electron transfer knockouts *Etf-QO*, and *CG7834*, both strains were homozygous lethal (findings summarized in Table 4).

We will discuss each of these genes further individually. MCAD deficiency is the most common inherited FAOD (Chang *et al.* 2024), caused by a disruption in the gene *ACADM*. Individuals with MCAD deficiency experience a wide range of symptom severity, including death, usually precipitated by fasting or increased energy requirements. Acylcarnitine profiles in humans with MCAD deficiency are characterized by elevations in C6, C8, and C10:1 acylcarnitines, with the predominant peak at C8 (Miller *et al*. 2021). In a mouse with *Acadm* knocked out, these 3 acylcarnitines were also elevated, but with higher C6 and C10:1 elevations than C8 in serum (Tolwani *et al*. 2005). Here in *Drosophila*, we observe that again these 3 acylcarnitines are elevated in flies with *Mcad* knocked out, with the greatest elevation in C6 (Fig. 4). This observation is consistent with the acylcarnitine profiles observed for a different *Mcad* knockout that we had made previously (Course *et al*. 2018). That mutant fly was made using a different CRISPR-Cas9 technique and resulted in a different deletion, showing that the finding is reproducible. While not surprising, this result validates our finding that the previously named *CG12262* is likely the ortholog of human *ACADM*, and acts as a kind of “positive control” for this study.

SCAD deficiency, due to its lack of severity, has been re-classified as a biochemical phenotype rather than a disease. That said, while some individuals remain asymptomatic, some infants do experience failure to thrive, hypotonia, and/or seizures. Again, there is a wide range of severity, at least partially influenced by the feeding and stress state of the individual (Wolfe *et al*. 2018). SCAD deficiency, caused by a disruption in the gene *ACADS*, is characterized biochemically in humans by an elevation in C4 acylcarnitine (Miller *et al*. 2021). A mouse model of SCAD deficiency arose spontaneously several decades ago, and was confirmed to have elevated C4, as well (Schiffer *et al*. 1989; Wood *et al*. 1989). More than 1 fly gene has been predicted to be the ortholog of *ACADS*, with the top 2 from DIOPT being *Arc42* and *CG4860* (Supplementary Table 1). *Arc42* received a higher score, but both are annotated on FlyBase as orthologous to *ACADS*, so we were interested in observing both. By comparing their acylcarnitine profiles, we could see that *Arc42* knockout flies exhibited the C4 elevation characteristic of humans and mice lacking functional SCAD, while *CG4860* knockout flies did not (Fig. 5). Instead, we observe a significantly lower level of C4 in starved *CG4860* knockout flies, which could indicate a role in SCAD synthesis. In addition, this is the only fly for which we observe a modest but significant elevation of C2, which could be related to a role in ketone utilization (Fig. 6).

*HADHA*, along with *HADHB*, encodes a protein involved in the enzymatic complex mitochondrial trifunctional protein (MTP, Fig. 1). Biallelic pathogenic variants in this gene lead to LCHAD deficiency and TFP deficiency in humans. Both of these deficiencies can result in a variety of symptomologies and severities (Prasun *et al.* 2022). In addition, carriers of pathogenic variants in either gene are at a high risk of HELLP (hemolysis, elevated liver enzymes, and low platelets) syndrome during pregnancy when carrying an affected fetus (Saudubray *et al.* 2016). The characteristic acylcarnitine profile in affected humans shows elevations in long-chain acylcarnitines C16, C18:2, C18:1, C18, C14–OH, C16–OH, and C18–OH. In mice, knockout of the orthologous gene *Hadha* is neonatal lethal and significantly elevated serum acylcarnitines were C14, C16:1, C16, C18:2, and C18:1 (Ibdah *et al*. 2001). Another mouse model in which a common pathogenic variant, G1528C, was knocked into the mice using CRISPR-Cas9 resulted in phenotypes relevant to LCHAD deficiency, including blood plasma elevations in C16:1, C16, C18:2, C18, C16–OH, C18:1–OH, and C18–OH.

In flies, when the putative orthologous gene, *Mtpα*, was knocked out using the ends-out gene targeting technique, flies were not early-stage lethal as the mouse was (Kishita, Tsuda, and Aigaki 2012). This study found significant increases in the relative amounts of virtually all saturated (C18–C10) and unsaturated (C18–C12) fatty acid acylcarnitines and hydroxyacylcarnitines (C18–C12) in these flies’ vs controls. We sought to recapitulate this data here with our method of CRISPR-Cas9 knockout, but our CRISPR-Cas9 led to the exact removal of 1 amino acid—a valine—encoded in exon 2 (Fig. 2d). This valine aligns with an isoleucine in the human protein (Supplementary Figs. 2 and 3); because they are both branched chain amino acids, they are considered to affect the protein structure similarly. Intriguingly, this missense mutation still affected the acylcarnitine profile: C14–OH and 18:2 were significantly elevated in the starvation condition (Fig. 7). This finding suggests that the single valine deletion had a small but significant effect on overall enzyme function.

In addition to the above genes involved in β-oxidation, we also knocked out 2 genes predicted to be involved in electron transfer: *Etf-QO* and *CG7834*. Intriguingly, both mutant strains were homozygous lethal. We did perform acylcarnitine analysis on the *Etf-QO* heterozygotes, and unsurprisingly found no significant results (Supplementary Table 2). *Etf-QO* is predicted to be the fly ortholog of human *ETFDH*, while *CG7834* is predicted to be the fly ortholog of human *ETFB*. Biallelic pathogenic variants in either cause MAD deficiency, which can exhibit a wide range of severity as in the β-oxidation disorders, but often more severe and including death in early infancy. The fact that knockout of either electron transfer gene caused homozygous lethality in our flies is therefore not inconsistent with the human deficiencies, in the sense that humans show severe symptomology. On the other hand, it is not typically lethal in humans until a stress event, which suggests that this loss of function is more deleterious in flies.

In *Etf-QO* mutants made previously using ethyl methanesulfonate, the flies were homozygous lethal, which is consistent with what we observed here (Alves *et al*. 2012). Similarly, in *C. elegans*, loss of the *ETFDH* ortholog, *let-721*, is maternal effect lethal (Chew *et al*. 2009). In zebrafish, loss of the ortholog *etfdh* leads to acylcarnitine elevations in C4, C5, C6, C8, C14, C16, and C18 (Song *et al*. 2009). This overlaps with characteristic profile of MAD deficiency in humans: unsaturated acylcarnitines C4–18, saturated C14–18, and C3-DC are all expected to be elevated (Miller *et al*. 2021). Less work has been done on models of *ETFB*. The fact that loss of the putative fly ortholog, *CG7834*, exhibits the same homozygous lethal phenotype as that of loss of *Etf-QO* is consistent, and suggests that this gene is involved in the same pathway. Conditional knockout fly models would need to be made to study the effects of knocking out these genes in adult flies.

Overall, this work summarizes the creation and characterization of several fly lines in which putative orthologs of human FAO genes have been mutated. We show that acylcarnitine analysis is an effective way to observe FAO changes in flies. With proper validation, flies are a good model for studying mitochondrial FAO and its related disorders. Robust animal models of these disorders, in turn, will enable the study of their progression, intervention, and perplexing variability.

## Supplementary Material

jkaf139_Supplementary_Data

## Data Availability

*Drosophila* stocks recovered in this study are available upon request. The data supporting the findings of this study are available within the article and Supplementary material. Raw data are provided in Supplementary Tables 1 and 2. Further inquiries can be directed to the corresponding author. Supplemental material available at G3 online.
