# Supplementary material for: Characterizing fatty acid oxidation genes in Drosophila
Source: G3 (Bethesda). 2025 Jun 16;15(8):jkaf139. doi: 10.1093/g3journal/jkaf139 (PMC12341901; doi:10.1093/g3journal/jkaf139)
Supplement: jkaf139_Supplementary_Data [file jkaf139_supplementary_data.zip › Figures_S1,_S2,_S3_G3-2025-405977.pdf]

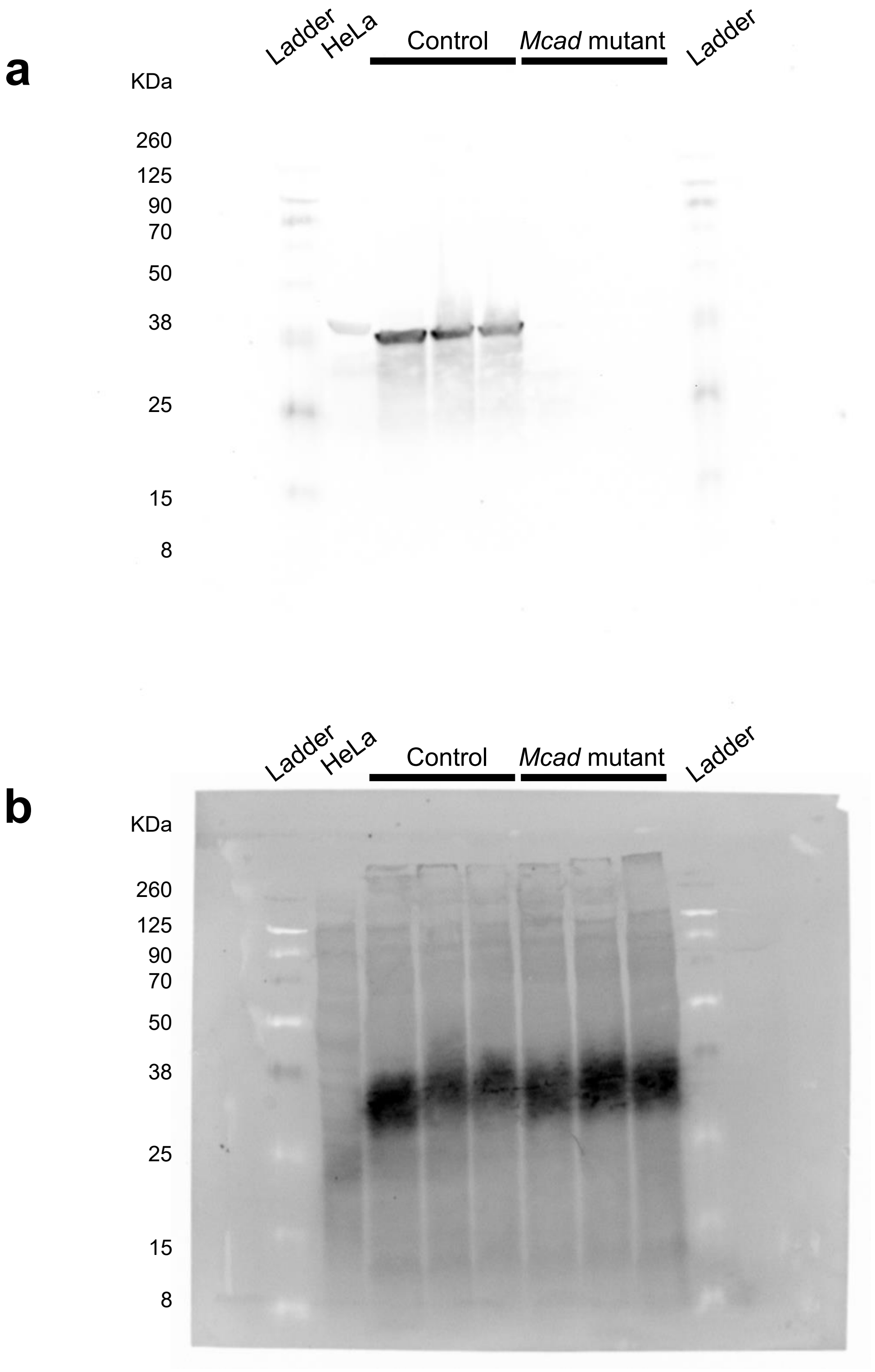

Figure S1. Raw images of (A) western blot and (B) total protein label shown in Figure 3. KDa is kilodaltons.

```
sp|P40939|ECHA_HUMAN      MVACRAIGILSRFSAFRILRSRGY-----------ICRNFTGSSALLTRTHINYGVKGDV     49
tr|Q9V397|Q9V397_DROME    MSATRFLSAVGQISRQQLLQNKCTRALPISAQLLQRRRLMSTNPAPVANKHLHTKVVNGV     60
                          * * * :. :.::*  ::*:.:               * :: . * ::..*::  * ..*

sp|P40939|ECHA_HUMAN      AVVRINSPNSKVNTLSKELHSEFSEVMNEIWASDQIRSAVLISSKPGCFIAGADINMLAA     109
tr|Q9V397|Q9V397_DROME    LVIKIDSPNAKVNSLGSEVSDEFERVIKDLETNPAVNSAVLISGKPGCFVAGADIGMLEA     120
                           *::*:***:***:*..*: .**..*:::: :.  :.******.*****:*****.** *

sp|P40939|ECHA_HUMAN      CKTLQEVTQLSQEAQRIVEKLEKSTKPIVAAINGSCLGGGLEVAISCQYRIATKDRKTVL     169
tr|Q9V397|Q9V397_DROME    CQTAEEATLISHGAQVMFDRMERSKKPIVAAISGVCLGGGLELALACHYRIATKDSKTKL     180
                          *:* :*.* :*: ** :.:::*:*.*******.* *******:*::*:******* ** *

sp|P40939|ECHA_HUMAN      GTPEVLLGALPGAGGTQRLPKMVGVPAALDMMLTGRSIRADRAKKMGLVDQLVEPLGPGL     229
tr|Q9V397|Q9V397_DROME    GLPEVMLGLLPGGGGTVRLPKLTSVPTALDMELTGKQVRADRAKRLGIVDLLVDPLGPGL     240
                          * ***:** ***.*** ****:..**:**** ***:.:******::*:** **:******

sp|P40939|ECHA_HUMAN      KPPEERTIEYLEEVAITFAKGLADKKISPKR-DKGLVEKLTAYAMTIPFVRQQVYKKVEE     288
tr|Q9V397|Q9V397_DROME    QPAEQNTIEYLEKTAVQVANDLASGKLRVNREKSGLVSKIQSFVMDTDFVKNKIFDTARK     300
                          :* *:.******:.*: .*:.**. *:  :* ..***.*: ::.*   **::::....:

sp|P40939|ECHA_HUMAN      KVRKQTKGLYPAPLKIIDVVKTGIEQGSDAGYLCESQKFGELVMTKESKALMGLYHGQVL     348
tr|Q9V397|Q9V397_DROME    QVLKASNGLYPAPLKILDVIRAGVDKGTDAGYEAERKGFGELSATPESKGLIALFRGQTE     360
                          :* * ::*********:**:::*:::*:**** .* : ****  * ***.*:.*::**.

sp|P40939|ECHA_HUMAN      CKKNKFGAPQKDVKHLAILGAGLMGAGIAQVSVDKGLKTILKDATLTALDRGQQQVFKGL     408
tr|Q9V397|Q9V397_DROME    CKKNRFGKPERPVKTVGVLGAGLMGAGIVQVSVDKGYQVVMKDATEAGLARGIGQVQKGL     420
                          ****:** *:: ** :.:**********.******* :.::**** :.* **  ** ***

sp|P40939|ECHA_HUMAN      NDKVKKKALTSFERDSIFSNLTGQLDYQGFEKADMVIEAVFEDLSLKHRVLKEVEAVIPD     468
tr|Q9V397|Q9V397_DROME    ETAVKRKRISALERDQTLASLRPTLDYSDFKNADIIIEAVFEDIKVKHRVIKELEAVVPE     480
                          :  **:* ::::***. ::.*   ***..*::**::*******:.:****:**:***:*:

sp|P40939|ECHA_HUMAN      HCIFASNTSALPISEIAAVSKRPEKVIGMHYFSPVDKMQLLEIITTEKTSKDTSASAVAV     528
tr|Q9V397|Q9V397_DROME    HCVIATNTSAIPITKIAAGSSRPEKVVGMHYFSPVDKMQLLEIITHPGTSKDTIAQAVAV     540
                          **::*:****:**::*** *.*****:****************   ***** *.****

sp|P40939|ECHA_HUMAN      GLKQGKVIIVVKDGPGFYTTRCLAPMMSEVIRILQEGVDPKKLDSLTTSFGFPVGAATLV     588
tr|Q9V397|Q9V397_DROME    GLKQGKVVITVGDGPGFYTTRILSTMLSEAIRLLQEGVDPKDLDQYTKKFGFPVGAATLA     600
                          *******:*.* ********* *: *:**.**:********.**. *..**********.

sp|P40939|ECHA_HUMAN      DEVGVDVAKHVAEDLGKVFGERFGGGNPELLTQMVSKGFLGRKSGKGFYIYQEGVKR-KD     647
tr|Q9V397|Q9V397_DROME    DEVGIDVGSHIAVDLAKAFGERFSGGNLEVMNDLVLAGFLGRKSGKGIFLYDGQKRGTRP     660
                          ****:**..*:* **.*.*****.*** *::.::*  **********::*:   :  :

sp|P40939|ECHA_HUMAN      LNSDMDSILASLKLPPKSEVSSDEDIQFRLVTRFVNEAVMCLQEGILATPAEGDIGAVFG     707
tr|Q9V397|Q9V397_DROME    VNNDALEIVKQKYALVSKGANAPEDLTLRMVSRFINEAVLCLEEKILDSPLEGDVGAVFG     720
                          :*.*  .*: .     .. ..: **: :*:*:**:****:**:* ** :* ***:*****

sp|P40939|ECHA_HUMAN      LGFPPCLGGPFRFVDLYGAQKIVDRLKKYEAAYGKQFTPCQLLADHANSPNKKFYQ----     763
tr|Q9V397|Q9V397_DROME    LGFPPFSGGPFRWVDQYGAGKLVSKMQSYAELYGAPFKPAQTLLDMAKDPSKKFYPKTGS     780
                          *****  *****:** *** *:*.:::.*   **  *.*.* * * *:.*.****

sp|P40939|ECHA_HUMAN      --- 763
tr|Q9V397|Q9V397_DROME    SKL 783
```

Figure S2. Alignment of protein products of human *HADHA* and fly *MTPα*, transcript A. *HADHA* encodes the protein Trifunctional enzyme subunit alpha, mitochondrial (P40939) and *MTPα* encodes the protein enoyl-CoA hydratase. There are two main isoforms produced from this gene; here is shown the product of transcript A, which is the longer isoform (Q9V397). Red highlights the amino acid deleted in the *MTPα* fly mutant; orange highlights the Clp/crotonase domain; blue highlights the NAD(P)-binding Rossmann-fold domain; and fuchsia highlights the 6-phosphogluconate dehydrogenase C-terminal domains. (*) is fully conserved residues (identical), (:) is functionally conserved residues (strongly similar properties), and (.) is partly functionally conserved residues (weakly similar properties).

```
sp|P40939|ECHA_HUMAN        MVACRAIGILSRFSAFRILRSRGYICRNFTGSSALLTRTHINYGVKGDVAVVRINSPNSK      60
tr|Q8IPE8|Q8IPE8_DROME      ----------------------------MSTNPAPVANKHLHTKVVNGVLVIKIDSPNAK      32
                                                        :: . * ::..*::  * ..* *::*:***:*

sp|P40939|ECHA_HUMAN        VNTLSKELHSEFSEVMNEIWASDQIRSAVLISSKPGCFIAGADINMLAACKTLQEVTQLS      120
tr|Q8IPE8|Q8IPE8_DROME      VNSLGSEVSDEFERVIKDLETNPAVNSAVLISGKPGCFVAGADIGMLEACQTAEEATLIS      92
                            **:*..*: .**..*:::: :.  :.******.*****:*****.** **:* :*.* :*

sp|P40939|ECHA_HUMAN        QEAQRIVEKLEKSTKPIVAAINGSCLGGGLEVAISCQYRIATKDRKTVLGTPEVLLGALP      180
tr|Q8IPE8|Q8IPE8_DROME      HGAQVMFDRMERSKKPIVAAISGVCLGGGLELALACHYRIATKDSKTKLGLPEVMLGLLP      152
                            : ** :.:::*:*.*******.* *******:*::*:******* ** ** ***:** **

sp|P40939|ECHA_HUMAN        GAGGTQRLPKMVGVPAALDMMLTGRSIRADRAKKMGLVDQLVEPLGPGLKPPEERTIEYL      240
tr|Q8IPE8|Q8IPE8_DROME      GGGGTVRLPKLTSVPTALDMELTGKQVRADRAKRLGIVDLLVDPLGPGLQPAEQNTIEYL      212
                            *.*** ****:..**:**** ***:.:******::*:** **:******:* *:.*****

sp|P40939|ECHA_HUMAN        EEVAITFAKGLADKKISPKR-DKGLVEKLTAYAMTIPFVRQQVYKKVEEKVRKQTKGLYP      299
tr|Q8IPE8|Q8IPE8_DROME      EKTAVQVANDLASGKLRVNREKSGLVSKIQSFVMDTDFVKNKIFDTARKQVLKASNGLYP      272
                            *:.*: .*:.**. *:  :* ..***.*: ::.*   **:::::....::* * ::****

sp|P40939|ECHA_HUMAN        APLKIIDVVKTGIEQGSDAGYLCESQKFGELVMTKESKALMGLYHGQVLCKKNKFGAPQK      359
tr|Q8IPE8|Q8IPE8_DROME      APLKILDVIRAGVDKGTDAGYEAERKGFGELSATPESKGLIALFRGQTECKKNRFGKPER      332
                            *****:**:::*:::*:**** .* : ****  * ***.*:.*::**. ****:** *::

sp|P40939|ECHA_HUMAN        DVKHLAILGAGLMGAGIAQVSVDKGLKTILKDATLTALDRGQQQVFKGLNDKVKKKALTS      419
tr|Q8IPE8|Q8IPE8_DROME      PVKTVGVLGAGLMGAGIVQVSVDKGYQVVMKDATEAGLARGIGQVQKGLETAVKRKRISA      392
                             ** :.:**********.******* :.::**** :.* **  ** ***:  **:* :::

sp|P40939|ECHA_HUMAN        FERDSIFSNLTGQLDYQGFEKADMVIEAVFEDLSLKHRVLKEVEAVIPDHCIFASNTSAL      479
tr|Q8IPE8|Q8IPE8_DROME      LERDQTLASLRPTLDYSDFKNADIIIEAVFEDIKVKHRVIKELEAVVPEHCVIATNTSAI      452
                            :***. ::.*   ***..*::**::*******:.:****:**:***:*:**::*:****:

sp|P40939|ECHA_HUMAN        PISEIAAVSKRPEKVIGMHYFSPVDKMQLLEIITTEKTSKDTSASAVAVGLKQGKVIIVV      539
tr|Q8IPE8|Q8IPE8_DROME      PITKIAAGSSRPEKVVGMHYFSPVDKMQLLEIITHPGTSKDTIAQAVAVGLKQGKVVITV      512
                            **::*** *.*****:******************   ***** *.***********:*.*

sp|P40939|ECHA_HUMAN        KDGPGFYTTRCLAPMMSEVIRILQEGVDPKKLDSLTTSFGFPVGAATLVDEVGVDVAKHV      599
tr|Q8IPE8|Q8IPE8_DROME      GDGPGFYTTRILSTMLSEAIRLLQEGVDPKDLDQYTKKFGFPVGAATLADEVGIDVGSHI      572
                             ********* *: *:**.**:********.**. *..**********.****:**..*:

sp|P40939|ECHA_HUMAN        AEDLGKVFGERFGGGNPELLTQMVSKGFLGRKSGKGFYIYQEGVKR-KDLNSDMDSILAS      658
tr|Q8IPE8|Q8IPE8_DROME      AVDLAKAFGERFSGGNLEVMNDLVLAGFLGRKSGKGIFLYDGQKRGTRPVNNDALEIVKQ      632
                            * **.*.*****.*** *::.::*  **********:::*:   :  : :*.*  .*: .

sp|P40939|ECHA_HUMAN        LKLPPKSEVSSDEDIQFRLVTRFVNEAVMCLQEGILATPAEGDIGAVFGLGFPPCLGGPF      718
tr|Q8IPE8|Q8IPE8_DROME      KYALVSKGANAPEDLTLRMVSRFINEAVLCLEEKILDSPLEGDVGAVFGLGFPPFSGGPF      692
                                 .. ..: **: :*:*:**:****:**:* ** :* ***:**********  ****

sp|P40939|ECHA_HUMAN        RFVDLYGAQKIVDRLKKYEAAYGKQFTPCQLLADHANSPNKKFYQ-------      763
tr|Q8IPE8|Q8IPE8_DROME      RWVDQYGAGKLVSKMQSYAELYGAPFKPAQTLLDMAKDPSKKFYPKTGSSKL      744
                            *:** *** *:*.:::.*   **  *.*.* * * *:.*.****
```

Figure S3. Alignment of protein products of human *HADHA* and fly *MTPα*, transcript B. *HADHA* encodes the protein Trifunctional enzyme subunit alpha, mitochondrial (P40939) and *MTPα* encodes the protein enoyl-CoA hydratase. There are two main isoforms produced from this gene; here is shown the product of transcript B, which is the shorter isoform (Q8IPE8). Red highlights the amino acid deleted in the *MTPα* fly mutant; orange highlights the Clp/crotonase domain; blue highlights the NAD(P)-binding Rossmann-fold domain; and fuchsia highlights the 6-phosphogluconate dehydrogenase C-terminal domains. (*) is fully conserved residues (identical), (:) is functionally conserved residues (strongly similar properties), and (.) is partly functionally conserved residues (weakly similar properties).
